# Renal function and efficacy of dual antiplatelet vs. alteplase in minor stroke: a *post hoc* analysis of ARAMIS study

**DOI:** 10.3389/fneur.2025.1568711

**Published:** 2025-04-30

**Authors:** Xiao-Yi He, Chao He, Hui-Sheng Chen

**Affiliations:** ^1^Dalian Medical University, Dalian, China; ^2^Department of Neurology, General Hospital of Northern Theater Command, Shenyang, China; ^3^Department of Neurology, Affiliated Zhongshan Hospital of Dalian University, Dalian, China

**Keywords:** clinical outcome, dual antiplatelet, intravenous thrombolysis, renal function, minor stroke

## Abstract

**Background:**

This secondary analysis of the ARAMIS trial evaluated renal function’s modifying effects on therapeutic responses to dual antiplatelet therapy (DAPT) versus intravenous thrombolysis in acute minor ischemic stroke.

**Methods:**

Based on the as-treated set, we stratified patients by admission estimated glomerular filtration rate into three groups: normal renal function (≥90 mL/min/1.73 m^2^), mildly decreased renal function (eGFR 60 to 89 mL/min/1.73 m^2^), and moderate to severe impairment renal function group (<60 mL/min/1.73 m^2^). The primary endpoint was excellent functional outcome defined as a modified Rankin Scale score of 0–1 at 90 days.

**Results:**

Among 615 analyzed patients, 367 (59.7%) exhibited normal renal function, 209 (34.0%) exhibited mildly decreased renal function and 39 (6.3%) exhibited moderate to severe impairment renal function. A numerically higher rate of excellent functional outcome was found in normal renal function patients with DAPT vs. alteplase (94.4% vs. 90.4%; *p* = 0.147), while no intergroup difference emerged in mildly decreased renal function patients (93.9% vs. 93.7%; *p* = 0.958) and moderate to severe impairment renal function patients (93.8% vs. 95.7%; *p* = 0.792). There was no significant interaction between treatment and renal function on the primary outcome (adjusted interaction *p* = 0.337).

**Conclusion:**

Among patients with normal renal function, DAPT was associated with a numerically higher, but not statistically significant, rate of excellent functional outcome in patients with minor nondisabling acute ischemic stroke presenting within 4.5 h of symptom onset compared with alteplase.

**Clinical trial registration:**

ClinicalTrials.gov, identifier NCT03661411.

## Introduction

Intravenous thrombolysis constitutes standard care for acute ischemic stroke, its clinical utility in minor nondisabling strokes remains a subject of clinical equipoise (1). Contemporary evidence demonstrates dual antiplatelet therapy’s (DAPT) capacity to mitigate stroke recurrence when initiated within 24 h of symptom onset (2, 3), though its functional outcome benefits remain unestablished. Recently, the ARAMIS (Antiplatelet vs. R-tPA for Acute Mild Ischemic Stroke) trial established noninferiority of DAPT versus alteplase for achieving 90-day functional independence (mRS 0–1) in minor strokes treated within 4.5 h (4).

Chronic kidney disease affects >30% of ischemic stroke patients (5), with emerging data implicating renal insufficiency as an independent predictor of stroke severity, post-stroke disability, and mortality (6–13). Further studies suggest that renal function modulates therapeutic efficacy across stroke interventions—influencing antiplatelet response (14), as well as thrombolysis (15) and thrombectomy outcomes (16). In addition, patients with renal dysfunction have increased platelet activation and aggregation, with a higher prevalence of poor responsiveness to aspirin or clopidogrel antiplatelet treatment (17–19). Despite these associations, the potential modification of DAPT versus thrombolysis treatment effects by renal status in minor stroke remains unexplored.

This investigation leverages ARAMIS trial data to elucidate renal function’s potential role as an effect modifier between DAPT and alteplase in minor nondisabling stroke, evaluating both therapeutic efficacy and safety profiles across renal function strata.

## Methods

### Study design and participants

The ARAMIS trial methodology has been comprehensively documented in primary publications (4), which was a multicenter randomized design comparing dual antiplatelet therapy (DAPT) against intravenous alteplase in minor stroke (NIHSS ≤ 5 with nondisabling deficits) treated ≤4.5 h post-onset. Inclusion required age ≥ 18 years with preserved consciousness (score = 0) and absence of clinically significant focal deficits (single-item NIHSS ≤ 1). Creatinine availability served as an exclusion criterion. Regulatory approvals were obtained from the General Hospital of Northern Theater Command ethics board and participating centers, with written informed consent from all participants.

### Renal stratification

Participants underwent renal function categorization using admission eGFR values: normal renal function group (≥90 mL/min/1.73 m^2^), mildly decreased renal function group (eGFR 60 to 89 mL/min/1.73 m^2^), and moderate to severe impairment renal function group (<60 mL/min/1.73 m^2^) (14, 20, 21). Therapeutic protocols included:

1. Thrombolysis arm: Weight-adjusted alteplase (0.9 mg/kg; 10% bolus + 90% infusion) capped at 90 mg, followed by standard antiplatelet regimens 24 h after alteplase.
2. DAPT arm: Clopidogrel loading (300 mg) on the first day and followed 75 mg/d (12 ± 2d) with aspirin (100 mg/d) followed by guideline-directed maintenance.
3. Neurological status was quantified via NIHSS at baseline and 24 h post-randomization. Follow-up evaluations at 90 days captured functional outcomes (mRS), vascular events, and safety endpoints.

### Calculation of eGFR

The eGFR was calculated using the Chronic Kidney Disease Epidemiology Collaboration creatinine equation (CKD-EPI) (22): eGFR = 141 × min(SCr/*k*,1)*^α^*× max(SCr/*k*,1)^–1.209^ × 0.993^Age^ × 1.018 (if female). Sex-specific coefficients: *k* = 0.7 (F)/0.9 (M); α = −0.329 (F)/−0.411 (M). The CKD-EPI China equation was calculated with a coefficient of 1.1 (23).

### Study outcomes

The primary outcome was excellent functional outcome at 90 days, defined as a modified Rankin Scale (mRS) score of 0 to 1. The secondary outcomes were favorable functional outcome (mRS score of 0 to 2) at 90 days, change in NIHSS score at 24 h, early neurological improvement at 24 h (defined as a decrease of 2 or more points in the NIHSS score), early neurological deterioration at 24 h (defined as an increase of 2 or more points in the NIHSS score but not as a result of cerebral hemorrhage), new stroke or other vascular events at 90 days, 90-day all-cause mortality, and ordinal shift of the mRS score at 90 days. The safety outcomes were symptomatic intracerebral hemorrhage (sICH), defined as evidence of bleeding on head computed tomographic scan associated with neurological deterioration (≥4-point increase in NIHSS score), and any bleeding event during the study.

### Statistical analysis

This exploratory secondary analysis was based on the as-treated set. Non-normal continuous variables expressed as median [IQR] with Mann–Whitney comparisons. Categorical data analyzed via χ^2^ tests.

For functional outcome at 90 days, change in NIHSS score at 24 h, early neurological improvement at 24 h, early neurological deterioration at 24 h, new stroke or other vascular events at 90 days, 90-day all-cause mortality, ordinal shift of mRS score at 90 days, sICH and any bleeding event, binary logistic regression analyses were performed. The treatment effects for the above outcomes are presented as odds ratios (ORs) with 95% confidence intervals (CIs). Covariate adjusted analyses were also performed for all outcomes, adjusting for the unbalanced baseline characteristics with *p* < 0.05. The interaction between renal function and treatment effect was also analyzed. As a sensitive analysis, we further compared the treatment effects in another grouping method (eGFR: ≥90 vs. <90 mL/min/1.73m^2^). SPSS 26.0 software and R software version 4.1.0 (R Foundation for Statistical Computing) were used for statistical analysis, and a bilateral test was used. When *p* < 0.05 there was statistical significance.

## Results

### Baseline characteristics

**Figure 1:**
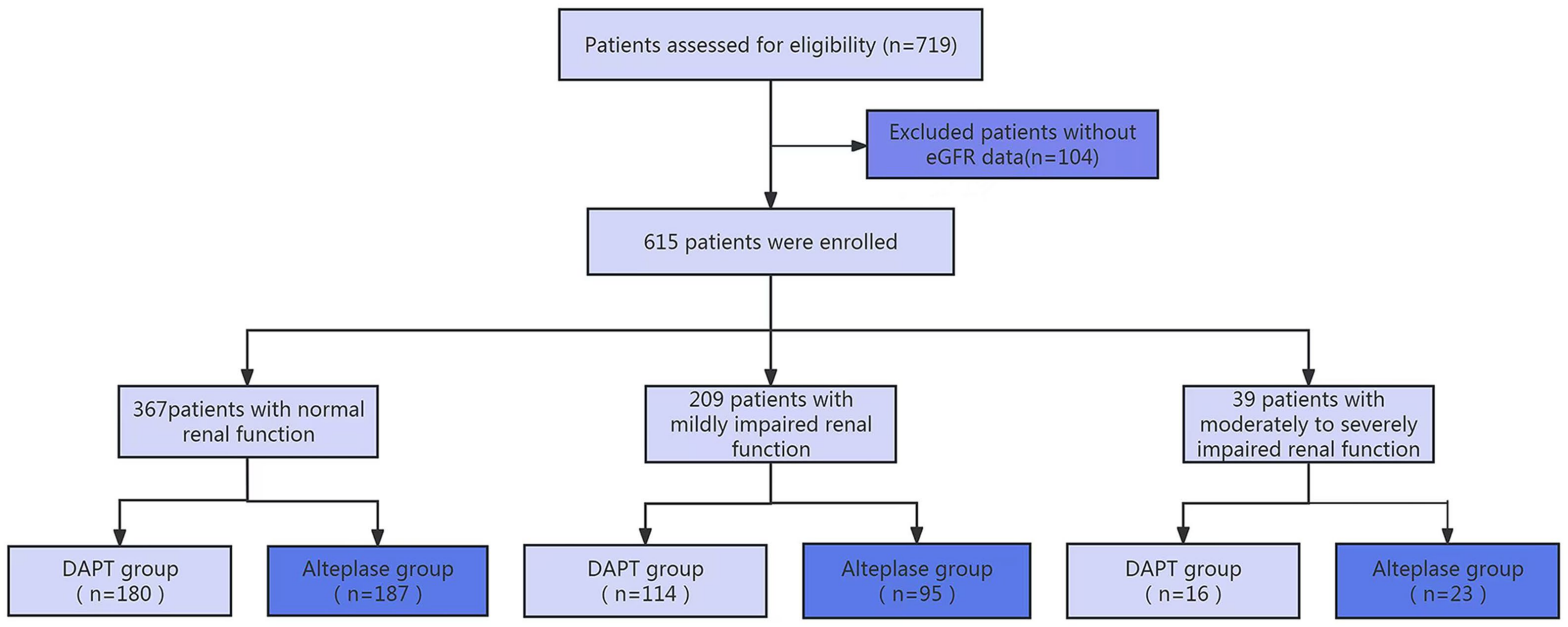
The flow chart of the study.

As shown in Figure 1, following exclusion of 104 participants lacking baseline eGFR measurements, the final cohort comprised 615 individuals stratified by renal function status: normal (*n* = 367, 59.7%) versus mildly decreased (*n* = 209, 34.0%) and moderate to severe impairment (*n* = 39, 6.3%) (Figure 1). Table 1 presented patient characteristics among three groups by eGFR category. Baseline demographic disparities emerged between renal subgroups for age, smoking history, and international normalized ratio. Table 2 compared the patient characteristics between DAPT and alteplase groups across the three groups. Treatment arms maintained balance across renal function categories except for sex and international normalized ratio in mildly decreased renal function subgroup, and systolic pressure in moderate to severe impairment renal function subgroup (Table 2).

**Table 1:** Comparison of patient characteristics among three groups by eGFR category.

Characteristics	Normal renal function (*n* = 367)	Mildly decreased renal function (*n* = 209)	Moderate to severe impairment renal function (*n* = 39)	*p* value
Age, y	60 (54–66)	70 (63–77)	69 (62–78)	<0.001
Sex				
Male	260 (70.8)	145 (69.4)	24 (61.5)	0.480
Female	107 (29.2)	64 (30.6)	15 (38.5)	
Current smoking^a^	142 (38.7)	61 (29.2)	5 (12.8)	0.001
Current drinking^a^	67 (18.3)	30 (14.4)	9 (23.1)	0.299
Medical history				
Hypertension	189 (51.5)	117 (56.0)	22 (56.4)	0.540
Diabetes	89 (24.3)	58 (27.8)	14 (35.9)	0.237
Previous stroke^b^	74 (20.2)	52 (24.9)	14 (35.9)	0.056
Previous TIA	1 (0.3)	2 (1.0)	0 (0)	0.424
Onset to treatment time, min	180 (129–226)	187 (139–230)	163 (120–217)	0.236
International normalized ratio	0.99 (0.93–1.04)	1.01 (0.95–1.06)	0.96 (0.91–1.02)	0.002
Activated partial thromboplastin time, s	31.7 (27.2–36.1)	33.0 (28.3–37.4)	32.3 (28.5–36.6)	0.050
Systolic blood pressure, mm Hg	150 (138–163)	150.0 (139–163)	160 (138–170)	0.242
Diastolic blood pressure, mm Hg	88 (80–97)	87 (80–93)	88 (80–98)	0.181
Blood glucose, mmol/L	6.6 (5.5–9.3)	6.5 (5.5–9.6)	7.4 (5.7–9.7)	0.403
Baseline NIHSS^c^	2 (1–3)	2 (1–3)	2 (1–3)	0.822
Estimated premorbid function (mRS)				
mRS 0	280 (76.3)	145 (69.4)	26 (66.7)	0.122
mRS 1	87 (23.7)	64 (30.6)	13 (33.3)	
Presumed stroke cause^d^				
Large artery atherosclerosis	56 (15.3)	34 (16.3)	4 (10.3)	0.595
Cardioembolic	1 (0.3)	0 (0)	1 (2.6)	
Small artery occlusion	93 (25.3)	47 (22.5)	10 (25.6)	
Other determined cause	2 (0.5)	2 (10.0)	0	
Undetermined cause	215 (58.6)	126 (60.3)	24 (61.5)	

The data was shown with median (interquartile range) or number (percentage). NIHSS, National Institutes of Health Stroke Scale (NIHSS); mRS, modified Rankin scale. ^a^Current smoking defined as consuming at least 1 cigarette per day within 1 year before the onset of the disease. Current drinking defined as consuming alcohol at least once a week within 1 year before the onset of the disease and consume alcohol continuously for more than 1 year. ^b^Referring only to patients with premorbid modified Rankin Scale score ≤ 1. ^c^Patients with NIHSS scores less than or equal to 5 were eligible for this study; NIHSS scores range from 0 to 42, with higher scores indicating more severe neurological deficit. ^d^The presumed stroke cause was classified according to the Trial of Org 10,172 in the Acute Stroke Treatment (TOAST) classification system.

**Table 2:** Comparison of patient characteristics between DAPT and alteplase groups across the three groups by eGFR category.

Characteristics	Normal renal function (*n* = 367)			Mildly decreased renal function (*n* = 209)			Moderate to severe impairment renal function (*n* = 39)		
	DAPT (*n* = 180)	Alteplase (*n* = 187)	*p* value	DAPT (*n* = 114)	Alteplase (*n* = 95)	*p* value	DAPT (*n* = 16)	Alteplase (*n* = 23)	*p* value
Age, y	60 (54–67)	60 (55–66)	0.439	69 (63–77)	71 (63–78)	0.446	71 (64–80)	68 (60–78)	0.310
Sex									
Male	122 (67.8)	138 (73.8)	0.205	86 (75.4)	59 (62.1)	0.037	9 (56.3)	15 (65.2)	0.571
Female	58 (32.2)	49 (26.2)		28 (24.6)	36 (37.9)		7 (43.8)	8 (34.8)	
Current smoking^a^	66 (36.7)	76 (40.6)	0.434	35 (30.7)	26 (27.4)	0.598	1 (6.3)	4 (17.4)	0.631
Current drinking^a^	32 (17.8)	35 (18.7)	0.563	20 (17.5)	10 (10.5)	0.150	2 (12.5)	7 (30.4)	0.262
Medical history									
Hypertension	100 (55.6)	89 (47.6)	0.127	69 (60.5)	48 (50.5)	0.147	10 (62.5)	12 (52.2)	0.522
Diabetes	44 (24.4)	45 (24.1)	0.932	36 (31.6)	22 (23.2)	0.176	6 (37.5)	8 (34.8)	0.862
Previous stroke^b^	36 (20.0)	38 (20.3)	0.939	26 (22.8)	26 (27.4)	0.448	6 (37.5)	8 (34.8)	0.862
Previous TIA	1 (0.6)	0 (0)	0.490	1 (0.9)	1 (1.1)	1.000	0 (0)	0 (0)	NA
OTT, min	184 (136–230)	168 (123–216)	0.061	184 (135–227)	188 (147–233)	0.389	163 (107–212)	188 (124–225)	0.710
INR	0.99 (0.92–1.04)	0.98 (0.94–1.05)	0.582	1.02 (0.96–1.08)	0.99 (0.93–1.04)	0.012	0.95 (0.90–1.05)	0.97 (0.91–1.00)	0.875
APTT, s	31.3 (27.0–36.1)	32.1 (27.4–36.1)	0.507	33.9 (28.2–38.7)	32.5 (28.4–36.1)	0.129	31.9 (27.7–38.6)	32.3 (28.5–36.6)	0.977
SBP, mm Hg	150 (136–166)	152 (138–161)	0.773	152 (138–167)	150 (140–161)	0.528	149 (131–166)	167 (145–174)	0.036
DBP, mm Hg	88 (81–95)	89 (80–98)	0.629	87 (80–94)	87 (80–93)	0.546	87 (77–96)	90 (82–98)	0.297
BG, mmol/L	6.6 (5.4–10.6)	6.6 (5.5–8.7)	0.916	6.6 (5.5–10.6)	6.2 (5.4–8.5)	0.292	7.2 (5.6–17.1)	7.4 (6.2–9.7)	0.786
Baseline NIHSS^c^	2 (1–3)	2 (1–3)	0.512	2 (1–3)	2 (2–3)	0.057	2 (1–3)	2 (1–3)	0.953
Estimated premorbid function (mRS)									
mRS 0	142 (78.9)	138 (73.8)	0.252	81 (71.1)	64 (67.4)	0.565	8 (50.0)	18 (78.3)	0.066
mRS 1	38 (21.1)	49 (26.2)		33 (28.9)	31 (32.6)		8 (50.0)	5 (21.7)	
Presumed stroke cause^d^									
LAA	28 (15.6)	28 (15.0)	0.737	20 (17.5)	14 (14.7)	0.943	1 (6.3)	3 (13.0)	0.879
Cardioembolic	1 (0.6)	0 (0)		0 (0)	0 (0)		0 (0)	1 (4.3)	
SAO	45 (25.0)	48 (25.7)		25 (21.9)	22 (23.2)		5 (31.3)	5 (21.7)	
ODC	0 (0)	2 (1.1)		1 (0.9)	1 (1.1)		0 (0)	0 (0)	
UC	106 (58.9)	109 (58.3)		68 (59.6)	58 (61.1)		10 (62.5)	14 (60.9)	

The data was shown with median (interquartile range) or number (percentage). Abbreviations: DAPT, dual antiplatelet therapy; TIA, transient ischemic attack; OTT, time from onset of symptoms to receipt of assigned treatment; INR, international normalized ratio; APTT, activated partial thromboplastin time; BG, blood glucose; SBP, systolic blood pressure; DBP, diastolic blood pressure; mRS, modified Rankin scale; LAA, large artery atherosclerosis; SAO, small artery occlusion; ODC, other determined cause; UC, undetermined cause. ^a^Current smoking defined as consuming at least 1 cigarette per day within 1 year before the onset of the disease. Current drinking defined as consuming alcohol at least once a week within 1 year before the onset of the disease and consume alcohol continuously for more than 1 year. ^b^Referring only to patients with premorbid modified Rankin Scale score ≤ 1. ^c^Patients with National Institutes of Health Stroke Scale (NIHSS) scores less than or equal to 5 were eligible for this study; NIHSS scores range from 0 to 42, with higher scores indicating more severe neurological deficit. ^d^The presumed stroke cause was classified according to the Trial of Org 10,172 in the Acute Stroke Treatment (TOAST) classification system.

**Figure 2:**
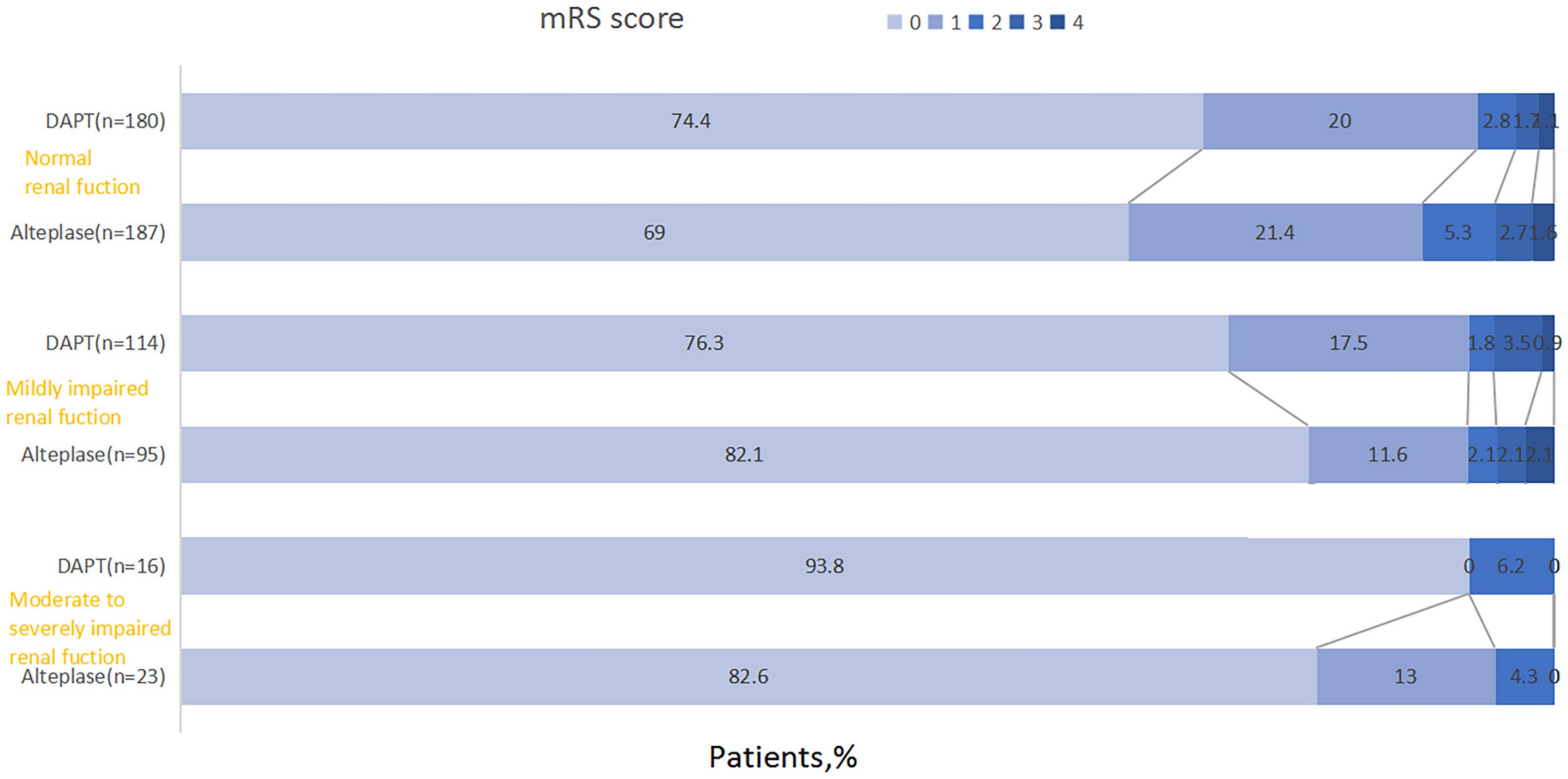
Distribution of modified Rankin Scale (mRS) score between three groups according to renal function status.

Table 3 presented clinical outcomes. Intervention effects stratified by renal function revealed a numerically higher proportion of excellent functional outcome (mRS 0–1) with DAPT versus alteplase in normal renal function (94.4% vs. 90.4%; absolute difference 4.0%, OR = 1.81 [95% CI 0.81–4.04], *p* = 0.147), contrasting with comparable efficacy in mildly decreased renal function subgroup (93.9% vs. 93.7%; absolute difference: 0.2%; adjusted OR = 1.095 [95% CI 0.343–3.494], *p* = 0.878) and moderate to severe impairment renal function subgroup (93.8% vs. 95.7%; absolute difference: 1.9%; adjusted OR = 0.536 [95% CI 0.025–11.342], *p* = 0.688) (Table 3). Formal interaction testing demonstrated nonsignificant renal function-by-treatment effect on primary outcome (adjusted P interaction = 0.337). As shown in Figure 2, parallel patterns emerged for functional recovery (mRS 0–2) and ordinal mRS distribution.

**Table 3:** Clinical outcomes stratified by eGFR category.

Outcome	Renal function	DAPT	Alteplase	Treatment effect metric	OR (95% CI)	*p* value	Adjusted^a^ OR (95% CI)	*p* value	Adjusted^b^ *p* interaction
mRS 0 to 1 at 90 days^c^	Normal	170 (94.4)	169 (90.4)	OR	1.811 (0.812–4.037)	0.147	/	/	0.337
	Mildly decreased	107 (93.9)	89 (93.7)		1.030 (0.334–3.178)	0.958	1.095 (0.343–3.494)	0.878	
	Moderate to severe impairment	15 (93.8)	22 (95.7)		0.682 (0.040–11.769)	0.792	0.536 (0.025–11.342)	0.688	
mRS 0 to 2 at 90 days^c^	Normal	175 (97.2)	179 (95.7)	OR	1.564 (0.502–4.874)	0.440	/	/	0.447
	Mildly decreased	109 (95.6)	91 (95.8)		0.958 (0.250–3.674)	0.950	0.899 (0.225–3.595)	0.881	
	Moderate to severe impairment	16 (100.0)	23 (100.0)		NA	NA	NA	NA	
mRS distribution at 90 days	Normal			OR	1.347 (0.857–2.117)	0.196	/	/	
	Mildly decreased				0.723 (0.368–1.420)	0.347	0.691 (0.345–1.384)	0.297	
	Moderate to severe impairment				2.930 (0.304–28.191)	0.352	2.995 (0.282–31.785)	0.363	
ENI within 24 h^d^	Normal	26 (14.4)	42 (22.5)	OR	0.583 (0.340–0.999)	0.050	/	/	0.518
	Mildly decreased	18 (15.8)	23 (24.2)		0.587 (0.295–1.168)	0.129	0.643 (0.316–1.310)	0.224	
	Moderate to severe impairment	2 (12.5)	3 (13.0)		0.952 (0.140–6.465)	0.960	0.788 (0.103–5.897)	0.808	
END within 24 h^e^	Normal	8 (4.4)	18 (9.6)	OR	0.437 (0.185–1.031)	0.059	/	/	0.536
	Mildly decreased	2 (1.8)	5 (5.3)		0.321 (0.061–1.696)	0.181	0.314 (0.059–1.679)	0.176	
	Moderate to severe impairment	1 (6.3)	4 (17.4)		0.317 (0.032–3.138)	0.326	0.622 (0.053–7.343)	0.706	
Change in NIHSS at 24 h^f^	Normal	0 (−0.75 to 0)	0 (−1.00 to 0)	GMR	0.057 (−0.368–0.482)	0.105	/	/	
	Mildly decreased	0 (−1.00 to 0)	0 (−1.00 to 0)		−0.054 (−0.440–0.331)	0.782	−0.049 (−0.439–0.340)	0.804	
	Moderate to severe impairment	−0.50 (−1.00 to 0)	0 (−1.00 to 0)		−0.660 (−1.872–0.551)	0.285	−0.414 (−1.673–0.845)	0.519	
Stroke or other vascular events within 90 days	Normal	0 (0)	2 (1.1)	HR	NA	0.996	/	/	0.997
	Mildly decreased	0 (0)	0 (0)		NA	NA	NA	NA	
	Moderate to severe impairment	0 (0)	0 (0)		NA	NA	NA	NA	
Death at 90 days	Normal	0 (0)	0 (0)	RD	NA	NA	/	NA	NA
	Mildly decreased	0 (0)	0 (0)		NA	NA	NA	NA	
	Moderate to severe impairment	0 (0)	0 (0)		NA	NA	NA	NA	
sICH^g^	Normal	0/180 (0)	0/187 (0)	RD	NA	NA	/	/	1.000
	Mildly decreased	0/114 (0)	1/95 (1.1)		NA	0.996	NA	0.996	
	Moderate to severe impairment	0/16 (0)	0 (0)		NA	NA	NA	NA	
Any bleeding events	Normal	2/180 (1.1)	14/187 (7.5)	OR	0.139 (0.031–0.620)	0.010	/	/	0.035
	Mildly decreased	1/114 (0.9)	2/95 (2.1)		0.412 (0.037–4.610)	0.592	0.388 (0.034–4.398)	0.444	
	Moderate to severe impairment	1/16 (6.25)	0 (0)		0.000	0.998	0.000	0.998	

DAPT, dual antiplatelet therapy; OR, odds ratio; mRS, modified Rankin Scale; ENI, early neurological improvement; END, early neurological deterioration; GMR, geometric mean ratio; HR, hazard ratio; RD, risk difference; sICH, symptomatic intracerebral hemorrhage. ^a^Adjusted for key prognostic covariates (sex, INR, SBP). ^b^Adjusted for key prognostic covariates (age, smoking consumption, INR). ^c^mRS scores range from 0 to 6: 0 = no symptoms, 1 = symptoms without clinically significant disability, 2 = slight disability, 3 = moderate disability, 4 = moderately severe disability, 5 = severe disability, and 6 = death. ^d^Early neurological improvement was defined as a decrease in NIHSS score of ≥ 2 between baseline and 24 h. ^e^Early neurological deterioration was defined as an increase in NIHSS score of ≥ 2 between baseline and 24 h, but not as a result of cerebral hemorrhage. ^f^NIHSS scores range from 0–42, with higher scores indicating greater stroke severity. The log (NIHSS + 1) was analyzed using a generalized linear model. ^g^Symptomatic intracerebral hemorrhage was defined as any evidence of bleeding on the head computed tomographic scan associated with clinically significant neurological deterioration (4-point increase in NIHSS score).

Consistent with primary trial findings (4), thrombolysis arm exhibited greater 24-h neurological improvement rates and lower neurological deterioration incidence compared to DAPT, irrespective of renal status (Table 3). Secondary endpoints including 24 h NIHSS change, 90-day vascular events, and mortality showed no intergroup differences (Table 3).

As to the safety outcomes, no sICH occurred in patients with normal renal function and moderate to severe impairment renal function subgroup, while 1 patient experienced sICH in the alteplase group with mildly decreased renal function subgroup (Table 3).

As a sensitive analysis, we analyzed the results in patients stratified by eGFR ≥90 vs. <90 mL/min/1.73m^2^. The baseline characteristics of this stratification population are shown in Table 4. The similar results were found in this grouping patients (Table 5).

**Table 4:** Comparison of patient characteristics between the two groups by eGFR ≥90 vs.<90 mL/min/1.73m^2^.

Characteristics	Normal renal function (*n* = 367)			Abnormal renal function (*n* = 248)		
	DAPT (*n* = 180)	Alteplase (*n* = 187)	*p* value	DAPT (*n* = 130)	Alteplase (*n* = 118)	*p* value
Age, y	60 (54–67)	60 (55–66)	0.439	70 (63–77)	71 (62–78)	0.673
Sex						
Male	122 (67.8)	138 (73.8)	0.205	95 (73.1)	74 (62.7)	0.080
Female	58 (32.2)	49 (26.2)		35 (26.9)	44 (37.3)	
Current smoking^a^	66 (36.7)	76 (40.6)	0.434	36 (27.7)	30 (25.4)	0.686
Current drinking^a^	32 (17.8)	35 (18.7)	0.563	22 (16.9)	17 (14.4)	0.587
Medical history						
Hypertension	100 (55.6)	89 (47.6)	0.127	79 (60.8)	60 (50.8)	0.116
Diabetes	44 (24.4)	45 (24.1)	0.932	42 (32.3)	30 (25.4)	0.233
Previous stroke^b^	36 (20.0)	38 (20.3)	0.939	32 (24.6)	34 (28.8)	0.455
Previous TIA	1 (0.6)	0 (0)	0.490	1 (0.8)	1 (0.8)	1.000
OTT, min	184 (136–230)	168 (123–216)	0.061	180 (135–222)	188 (139–231)	0.418
INR	0.99 (0.92–1.04)	0.98 (0.94–1.05)	0.582	1.02 (0.95–1.08)	0.98 (0.93–1.04)	0.605
APTT, s	31.3 (27.0–36.1)	32.1 (27.4–36.1)	0.507	33.7 (28.2–38.7)	32.4 (28.5–36.1)	0.929
BG, mmol/L	6.6 (5.4–10.6)	6.6 (5.5–8.7)	0.916	6.7 (5.5–10.6)	6.6 (5.5–8.9)	0.005
SBP, mmHg	150 (136–166)	152 (138–161)	0.773	152 (138–166)	152 (140–165)	0.521
DBP, mmHg	88 (81–95)	89 (80–98)	0.629	87 (80–95)	87 (80–94)	0.804
Baseline NIHSS^c^	2 (1–3)	2 (1–3)	0.512	2 (1–3)	2 (1–3)	0.093
Estimated premorbid function (mRS)						
mRS 0	142 (78.9)	138 (73.8)	0.252	89 (68.5)	82 (69.5)	0.861
mRS 1	38 (21.1)	49 (26.2)		41 (31.5)	36 (30.5)	
Presumed stroke cause^d^						
LAA	28 (15.6)	28 (15.0)	0.737	21 (16.2)	17 (14.4)	0.950
Cardioembolic	1 (0.6)	0 (0)		0 (0)	1 (0.8)	
SAO	45 (25.0)	48 (25.7)		30 (23.1)	27 (22.9)	
ODC	0 (0)	2 (1.1)		1 (0.8)	1 (0.8)	
UC	106 (58.9)	109 (58.3)		78 (60.0)	72 (61.0)	

The data was shown with median (interquartile range) or number (percentage). Abbreviations: DAPT, dual antiplatelet therapy; TIA, transient ischemic attack; OTT, time from onset of symptoms to receipt of assigned treatment; INR, international normalized ratio; APTT, activated partial thromboplastin time; BG, blood glucose; SBP, systolic blood pressure; DBP, diastolic blood pressure; mRS, modified Rankin scale; LAA, large artery atherosclerosis; SAO, small artery occlusion; ODC, other determined cause; UC, undetermined cause. ^a^Current smoking defined as consuming at least 1 cigarette per day within 1 year before the onset of the disease. Current drinking defined as consuming alcohol at least once a week within 1 year before the onset of the disease and consume alcohol continuously for more than 1 year. ^b^Referring only to patients with premorbid modified Rankin Scale score ≤ 1. ^c^Patients with National Institutes of Health Stroke Scale (NIHSS) scores less than or equal to 5 were eligible for this study; NIHSS scores range from 0 to 42, with higher scores indicating more severe neurological deficit. ^d^The presumed stroke cause was classified according to the Trial of Org 10,172 in the Acute Stroke Treatment (TOAST) classification system.

**Table 5:** Clinical outcomes stratified by eGFR ≥ 90 vs. <90 mL/min/1.73m^2^.

Outcome	Renal function	DAPT	Alteplase	Treatment effect metric	Unadjusted		Adjusted^a^		Adjusted^b^ *p* interaction
					treatment difference (95% CI)	*p* value	treatment difference (95% CI)	*p* value	
mRS 0 to 1 at 90 d^c^	Normal	170 (94.4)	169 (90.4)	OR	1.811 (0.812–4.037)	0.147	/	/	0.357
	Abnormal	122 (93.8)	111 (94.1)		0.962 (0.338–2.738)	0.942	0.959 (0.331–2.776)	0.938	
mRS 0 to 2 at 90 d^c^	Normal	175 (97.2)	179 (95.7)	OR	1.564 (0.502–4.874)	0.440	/	/	0.566
	Abnormal	125 (96.2)	114 (96.6)		0.877 (0.230–3.347)	0.848	0.928 (0.236–3.654)	0.915	
mRS distribution at 90 d	Normal			OR	1.347 (0.857–2.117)	0.196	/	/	
	Abnormal				0.801 (0.428–1.498)	0.487	0.790 (0.418–1.492)	0.467	
ENI within 24 h^d^	Normal	26 (14.4)	42 (22.5)	OR	0.583 (0.340–0.999)	0.050	/	/	0.862
	Abnormal	20 (15.4)	26 (22.0)		0.643 (0.337–1.227)	0.180	0.565 (0.288–1.107)	0.096	
END within 24 h^e^	Normal	8 (4.4)	18 (9.6)	OR	0.437 (0.185–1.031)	0.059	/	/	0.599
	Abnormal	3 (2.3)	9 (7.6)		0.286 (0.076–1.083)	0.065	0.268 (0.068–1.055)	0.060	
Change in NIHSS at 24 h^f^	Normal	0 (−0.75 to 0)	0 (−1.00 to 0)	GMR	0.057 (−0.368–0.482)	0.792	/	/	
	Abnormal	0 (−1.00 to 0)	0 (−1.00 to 0)		−0.191 (−0.570–0.188)	0.324	−0.186 (−0.572–0.200)	0.345	
Stroke or other vascular events within 90 d	Normal	0 (0)	2 (1.1)	HR	NA	0.996	/	/	0.997
	Abnormal	0 (0)	0 (0)		NA	NA	NA	NA	
Death at 90 d	Normal	0 (0)	0 (0)	RD	NA	NA	/	/	NA
	Abnormal	0 (0)	0 (0)		NA	NA	NA	NA	
sICH^g^	Normal	0/180 (0)	0/187 (0)	RD	NA	NA	/	/	0.997
	Abnormal	0/130 (0)	1/118 (0.8)		NA	1.000	NA	0.996	
Any bleeding events	Normal	2/180 (1.1)	14/187 (7.5)	OR	7.202 (1.613–32.159)	0.010	/	/	0.117
	Abnormal	2/130 (1.5)	2/118 (1.7)		1.103 (0.153–7.960)	0.922	1.040 (0.143–7.562)	0.969	

DAPT, dual antiplatelet therapy; OR, odds ratio; GMR, geometric mean ratio; HR, hazard ratio; RD, risk difference; mRS, modified Rankin Scale; ENI, early neurological improvement; END, early neurological deterioration; sICH, symptomatic intracerebral hemorrhage. ^a^Adjusted for key prognostic covariates (blood glucose). ^b^Adjusted for key prognostic covariates (age, smoking consumption, DBP, mRS). ^c^mRS scores range from 0 to 6: 0 = no symptoms, 1 = symptoms without clinically significant disability, 2 = slight disability, 3 = moderate disability, 4 = moderately severe disability, 5 = severe disability, and 6 = death. There was 1 patient with disagreement over mRS (in the control group) between the central adjudicator and site assessor. ^d^Early neurological improvement was defined as a decrease in NIHSS score of ≥2 between baseline and 24 h. ^e^Early neurological deterioration was defined as an increase in NIHSS score of ≥2 between baseline and 24 h, but not as a result of cerebral hemorrhage. ^f^NIHSS scores range from 0 to 42, with higher scores indicating greater stroke severity. The log (NIHSS + 1) was analyzed using a generalized linear model. ^g^Symptomatic intracerebral hemorrhage was defined as any evidence of bleeding on the head computed tomographic scan associated with clinically significant neurological deterioration (4-point increase in NIHSS score).

**Figure 3:**
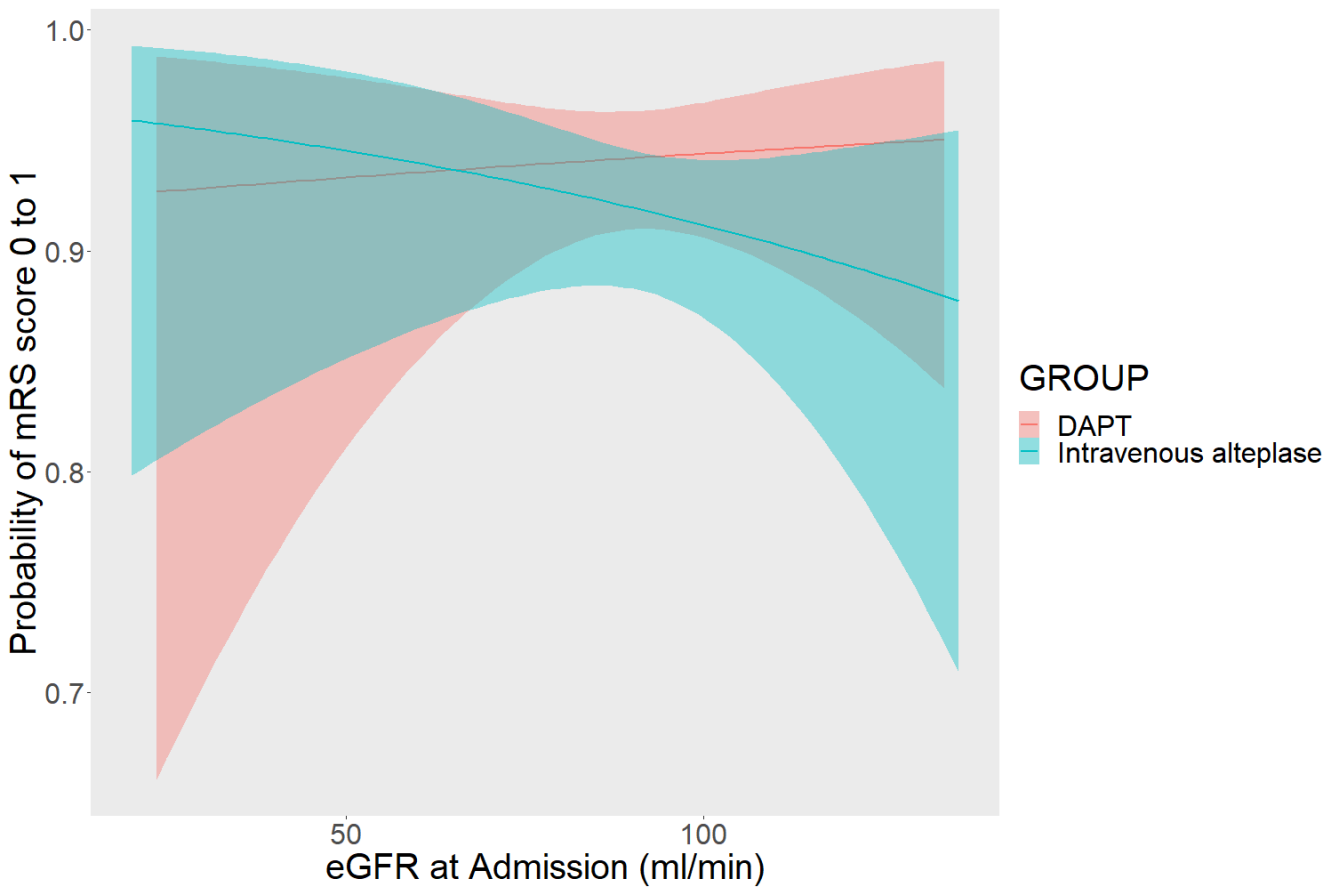
The relationship between the probability of modified Rankin Scale (mRS) score 0 to1 and estimated glomerular filtration rate (eGFR).

When eGFR was used as a continuous variable, DAPT was more likely to result in an excellent functional outcome than alteplase in patients with normal renal function (OR = 1.448 [95% CI 0.773–2.713], *p* = 0.247). As shown in Figure 3, the likelihood of an excellent functional outcome increased as eGFR increased in the DAPT group, but there was a reverse trend in the alteplase group.

## Discussion

The ARAMIS trial established non-inferiority of dual antiplatelet therapy (DAPT) versus intravenous alteplase for achieving 90-day functional independence in minor nondisabling strokes treated within 4.5 h. Our exploratory analysis revealed a numerical trend favoring DAPT over thrombolysis in preserved renal function cohorts (adjusted OR = 1.81), though statistical significance remained elusive potentially due to subgroup sample constraints.

In agreement with the previous epidemiological statistics (24), patients with renal impairment were older and had a higher burden of current smoking in this *post hoc* analysis. These results seem plausible given the obvious influence of age (25), and smoking (26)on renal function, and their close associations with stroke (27).

In this secondary analysis, we found that patients with better renal function are more likely to have an excellent functional outcome in DAPT group, which is consistent with previous findings that renal function was associated with the antiplatelet treatment effect after stroke (14). Nephropathy was associated with altered cerebral perfusion, cerebral neurovascular coupling, and blood vessel integrity (28). Furthermore, the kidney and brain share similar microvasculature and vaso-regulation, which makes them susceptible to microvascular dysfunction (29). These findings indicate that renal function may mirror cerebral autoregulation function (30). Collectively, we argue that impaired renal function may weaken the treatment effect of DAPT through impairing cerebral autoregulation, remodeling the cerebral vasculature, and reducing cerebral blood flow (CBF) (11). Similar to this phenomenon, previous studies have shown that patients with renal impairment did not benefit from intravenous thrombolysis treatment (31). One possible explanation could be that patients with renal insufficiency have reduced fibrinolysis rates, which may be due to less clot permeability and higher clot rigidity (32, 33). In addition, potential mechanisms by which renal impairment affects outcome and complications of intravenous thrombolysis may involve renal anemia, oxidative stress, inflammation, endothelial dysfunction, and paradoxical effects on hemostatic abnormalities, including increased risk of both bleeding and thrombosis (34, 35).

The major strength of this study was the first report to investigate the effect of renal function on the efficacy and safety of DAPT vs. alteplase based on a multicenter, randomized, open-label, blinded end-point assessment, noninferiority study. However, we admitted several limitations. The main limitation was the sample imbalance between two groups, which may weaken the statistical power as well as the validity of the findings. For example, moderate to severe impairment group only has 39 patients (6.3%). Second, only Chinese individuals were enrolled in this study, which may affect the generalizability of this finding given the differences in co-morbid factors and stroke etiology compared with other populations. Third, high rates of the primary outcome due to mild neurological deficit may have created a ceiling effect that limited the opportunity for either agent of DAPT or alteplase to show superiority to the other. These limitations would affect the generalizability of this finding. Finally, this finding should be interpreted with caution due to the nature of the secondary analysis.

In conclusion, among patients with normal renal function, DAPT was associated with a numerically higher, but not statistically significant, rate of excellent functional outcome at 90 days in patients with minor nondisabling acute ischemic stroke presenting within 4.5 h of symptom onset, compared with alteplase.

## Data Availability

The raw data supporting the conclusions of this article will be made available by the authors, without undue reservation.
